# Potential Health Impacts of Heavy Metals on HIV-Infected Population in USA

**DOI:** 10.1371/journal.pone.0074288

**Published:** 2013-09-02

**Authors:** Xiaohui Xu, Hui Hu, Amy B. Dailey, Greg Kearney, Evelyn O. Talbott, Robert L. Cook

**Affiliations:** 1 Department of Epidemiology, College of Public Health and Health Professions and College of Medicine, University of Florida, Gainesville, Florida, United States of America; 2 Health Sciences Department, Gettysburg College, Gettysburg, Pennsylvania, United States of America; 3 Department of Public Health, Brody School of Medicine, East Carolina University, Greenville, North Carolina, United States of America; 4 Department of Epidemiology, Graduate School of Public Health, University of Pittsburgh, Pittsburgh, Pennsylvania, United States of America; Stony Brook University, Graduate Program in Public Health, United States of America

## Abstract

**Purpose:**

Noninfectious comorbidities such as cardiovascular diseases have become increasingly prevalent and occur earlier in life in persons with HIV infection. Despite the emerging body of literature linking environmental exposures to chronic disease outcomes in the general population, the impacts of environmental exposures have received little attention in HIV-infected population. The aim of this study is to investigate whether individuals living with HIV have elevated prevalence of heavy metals compared to non-HIV infected individuals in United States.

**Methods:**

We used the National Health and Nutrition Examination Survey (NHANES) 2003-2010 to compare exposures to heavy metals including cadmium, lead, and total mercury in HIV infected and non-HIV infected subjects.

**Results:**

In this cross-sectional study, we found that HIV-infected individuals had higher concentrations of all heavy metals than the non-HIV infected group. In a multivariate linear regression model, HIV status was significantly associated with increased blood cadmium (p=0.03) after adjusting for age, sex, race, education, poverty income ratio, and smoking. However, HIV status was not statistically associated with lead or mercury levels after adjusting for the same covariates.

**Conclusions:**

Our findings suggest that HIV-infected patients might be significantly more exposed to cadmium compared to non-HIV infected individuals which could contribute to higher prevalence of chronic diseases among HIV-infected subjects. Further research is warranted to identify sources of exposure and to understand more about specific health outcomes.

## Introduction

Human Immunodeficiency Virus (HIV) infection is one of the most prevalent chronic infectious diseases, causing significant morbidity and mortality worldwide. An estimated 1,178,350 persons aged 13 and older were living with HIV infection in 2008 in the United States (U.S.) [1], and approximately 35 million people are currently living with HIV infection worldwide [2]. After the introduction and advancement of antiretroviral therapy (ART), HIV infections can now be managed for years, increasing the average life span of HIV infected individuals tremendously [3]. From 1996 to 2005, the average life expectancy after HIV diagnosis in the United States increased from 10.5 to 22.5 years [4], and the annual death rates of people with HIV have fell from 1.69% in 1999-2000 to 0.96% in 2007-2008 [5].

Together with these improvements in health outcomes for individuals living with HIV, noninfectious comorbidities including cardiovascular disease, hypertension, diabetes mellitus, bone fractures, renal failure, and HIV-related malignancies have become emerging health problems among this population today [6]. Noninfectious comorbidities now account for more than half of the deaths in recent years among HIV-infected patients who took ART [7,8]. Therefore, it is important to study the determinants of chronic diseases in this population.

Not only do HIV-infected patients have higher rates of cardiovascular disease than the general population but these outcomes also occur at a younger age, which could be due to chronic inflammation related to HIV infection and the use of antiretroviral medications [9,10]. Exposure to higher levels of environmental pollutants such as heavy metals, which have been reported to increase the risk of many chronic diseases in the general population [11–13], may explain earlier onset and/or the excess risks of chronic disease observed in HIV-infected individuals. Heavy metals including lead, cadmium, and mercury are widespread environmental exposures, and they ranked in the top 10 on the current Agency for Toxic Substances and Disease Registry Priority List of Hazardous Substances [14]. Exposure to cadmium could occur through consumption of shellfish and dark, leafy vegetable and cigarette smoking [15]. Lead exposure is totally due to inhalation of lead dust or ingestion of lead-contaminated food and water [16]. Mercury exposure occurs predominantly through consumption of fish [17]. The cumulative deleterious effects of these heavy metals can cause chronic degenerative changes [18], leading to cardiovascular, respiratory, liver, kidney, and nervous system diseases [19,20]. However, their potential health effects in HIV-infected group received little attention.

HIV-infected populations generally have a lower socio-economic status and live in poorer communities [21], which may consequently result in higher exposures to these toxins, considering the correlation between area-level poverty and environmental pollution. Moreover, HIV infection itself could increase sensitivity to the adverse health effects of environmental pollution because of the combination of the effects of environmental exposures and chronic inflammation due to HIV infection [22]. If HIV-infected patients are also at higher risk of exposure to these environmental pollutants compared to the general population, these exposures could contribute to the higher rates and/or the more rapid onset of noninfectious comorbidities among HIV-infected patients. To our knowledge, few studies have compared the levels of exposures to environmental heavy metals between HIV-infected and HIV-uninfected populations.

In this study, we analyzed the data from the 2003-2010 National Health and Nutrition Examination Surveys to examine HIV-infected patients’ exposures to three environmental pollutants, which were measured by blood cadmium, blood lead, and blood total mercury concentrations, compared to individuals without HIV infection.

## Methods

### 1.1 Study population

NHANES uses a complex, multistage sampling design to gain nationally representative samples of the non-institutionalized U.S. civilian population [23]. The NHANES surveys collect data from personal interviews, examinations, and laboratory tests of biological samples. The data have been released for public use in two-year increments since 1999. For NHANES 2003-2010, there were 51,838 persons selected for the biannual samples combined, 41,156 (79.4 percent) of those were interviewed, and 39,608 (76.4 percent) of those underwent a physical examination in a Mobile Examination Center (MEC) [24].

Blood cadmium, blood lead, and blood total mercury concentrations were measured simultaneously in a subsample (n=37,761) of persons 1 year and older in each biannual sample from 2003 to 2010. HIV antibody tests were done in a subsample (n=13,605) of persons 18 years old to 49 years old in the 2003-2008 data cycles and 18 years old to 59 years old in the 2009-2010 data cycle. For the present analysis, we selected all participants aged 18 to 49 years old who had HIV antibody tests and blood cadmium, lead and mercury tests during these four data cycles as the study population. Therefore, a total of 11,761 eligible participants were examined in this cross-sectional study.

Because each NHANES data cycle subsample was representative of the total population, the appropriate weight was calculated for the combined subsamples after considering the additional stage of sampling, the unequal probability of selection and the non-response rate [23].

### 1.2 Blood cadmium, lead and total mercury measurements

The laboratory procedure and quality control of blood cadmium, lead and total mercury concentrations are described in detail in the NHANES Laboratory/Medical Technologist Procedures Manual (LPM) [23]. Briefly, whole blood specimens were processed, frozen and then shipped to National Center for Environmental Health for testing. Blood cadmium, lead, and total mercury concentrations were measured by inductively coupled plasma mass spectrometry, based on quadrupole ICP-MS technology [25]. The coefficient of variations (CVs) range from 2.9%–11.3% for cadmium, 1.2%-4.4 for lead, and 2.3%-41.2% for total mercury through the study period.

The detection limit for cadmium, lead, and mercury in blood specimens are based on three times the standard deviation of blood blank run for a minimum of 20 runs [25]. The limit of detection of cadmium was 0.20µg/L throughout all data cycles. The limit of detection of lead was 0.3µg/dL (2003-2004 data cycles) and 0.25µg/dL (2005-2010 data cycles). The limit of detection of blood total mercury was 0.20ug/L (2003-2006 data cycle), 0.28µg/L (2007-2008 data cycles), and 0.33µg/L (2009-2010 data cycle). As recommended by the NHANES, when the result was below the limit of detection, the value for that variable is the detection limit divided by the square root of two.

### 1.3 HIV antibody test

As described detail in the NHANES LPM, blood specimens were processed, stored, and shipped to National Centers for Disease Control and Prevention [23]. All blood samples were tested using enzyme immunoassay (EIA) to detect antibody to human immunodeficiency virus type 1 or type 2 (Bio-Rad Laboratories, Hercules, CA). Positive EIA samples were retested with the same assay and repeatedly reactive samples were then tested by HIV-1 Western Blot (Calypte Biomedical Corporation, Rockville, MD). Based on the EIA and Western Blot results, samples were classified as HIV antibody positive, negative or indeterminant according to different patterns presented [26]. Subjects with positive HIV antibody test results were categorized as an HIV case, while those with negative results were categorized as a non-HIV case. Subjects with indeterminant HIV antibody test results were excluded from this study.

### 1.4 Serum cotinine measurements

Serum cotinine, which is a major metabolite of nicotine and has a half-life about 15-20 hours [27,28], was measured by the ID HPLC-APCI MS/MS method as described in the NHANES LPM in the combined subsample (n=35,232) of persons 3 years and older in each cycle from 2003–2010 [23]. Cotinine concentrations were derived from the ratio of native to labeled cotinine in the sample by comparisons to a standard curve. The limit of detection of serum cotinine was 0.015ng/mL throughout the study period. In cases, where the result was below the limit of detection, the value for that variable is the detection limit divided by the square root of two (i.e. 0.011ng/mL).

### 1.5 Other Covariates

Several covariates and potential confounders were included in this study. Age (18–49), gender, race/ethnicity (categorized as Non-Hispanic white, Non-Hispanic black, or others), and educational levels (categorized as less than high school, high school, or more than high school) were obtained by self-reported questionnaire. NHANES also calculated a poverty-to-income ratio (PIR). It was categorized as less than 1.0, 1.0-2.0, or more than 2.0 with the values < 1.00 as below the official poverty threshold [29]. Pack-year was calculated by multiplying the number of packs of cigarettes smoked per day by the number of years the person has smoked (i.e. 1 pack=20 cigarettes). All information on tobacco use was collected using self-reported questionnaires.

### 1.6 Statistical analysis

Descriptive statistics such as two-sided Student t-tests and Wald chi-square analysis were performed, where appropriate, after considering the complex sampling strategy. Results from all three heavy metals were log transformed to allow a comparison by the HIV antibody status. Multivariate linear regression models were used to evaluate the associations between HIV status and each heavy metal. We firstly used a model adjusting for covariates including age, sex, race/ethnicity, education, poverty income ratio, and serum cotinine, and a second model which additionally adjusted for pack-years, was also used. Since the outcome, heavy metals, were log-transformed in the analyses, we used exponential function of the point estimates obtained from the models for interpretations (i.e. the point estimate = log (Heavy metals for HIV-infected) – log (Heavy metal for non-HIV infected)). The sample weights, stratification and clustering design variables were also incorporated into all SAS survey procedures to ensure the correct estimation of sampling error. An 8-year MEC subsample weight was calculated for the combined 2003-2010 data by following the NHANES analytic and reporting guidelines and by assigning one-fourth of each 2-year MEC subsample weight 2003 to 2010 data cycles [30]. This calculated weight was used to analyze the combined NHANES 2003-2010 data. All statistical analyses were performed using SAS 9.3 survey procedures software (SAS Institute Inc., Cary, NC).

The protocol of NHANES has been approved by the Center for Disease Control and Prevention National Center of Health Statistics Research Ethics Review Board and this analysis was approved by the Institutional Review Board at the University of Florida.

## Results

Among 11,761 participants, 60 were HIV-infected subjects from the 2003-2010 NHANES data. Table 1 presents the geometric means of blood heavy metals according to HIV antibody status. Subjects with HIV had significantly higher levels of blood cadmium (0.47 vs. 0.33 ug/l, p=0.005) and lead (1.43 vs. 1.11 ug/l, p=0.016) compared to those without HIV. In addition, HIV-infected individuals had higher but not statistically significant different levels of total mercury (1.04 vs. 0.91 ug/l, p= 0.5) than HIV-uninfected population.

**Table 1:** Difference of Geometric Means of Blood Heavy Metal Concentration by HIV Status among US Adults (n=11761).

Heavy Metals	HIV			Non-HIV			p-value^b^
	N	<LOD^a^	Weighted Geometric Mean (95%CI)	N	<LOD^a^	Weighted Geometric Mean (95%CI)	
Blood Cadmium (ug/l)	60	12	0.47 (0.38-0.59)	11701	3177	0.34 (0.33-0.35)	<0.01
Blood Lead (ug/dl)	60	0	1.43 (1.17-1.75)	11701	46	1.11 (1.09-1.14)	0.02
Blood Total Mercury(ug/l)	60	7	1.04 (0.69-1.55)	11701	1588	0.91 (0.86-0.96)	0.50

^a^ LOD: Limit of detection.

^b^ p-value obtained from t-test after accounting for the complex survey design.

Table 2 presents the distribution of demographic and socioeconomic status by HIV status. Subjects with HIV infection were more likely to be male, older, non-Hispanic Black, and with lower PIR (all p<0.05). No significant differences in education were observed between HIV-infected persons and those without HIV infection.

**Table 2:** Distribution of demographic and socioeconomic status by HIV status (n=11,761).

Characteristics	HIV (n=60)		Non-HIV (n=11,701)		p-value
	N	Weighted Percent (95%CI)	N	Weighted Percent (95%CI)	
*Sex (%)*					
Male	47	77.05 (65.28-88.83)	5530	49.31 (48.40-50.22)	<0.01
Female	13	22.95 (11.17-34.72)	6171	50.69 (49.78-51.60)	
*Age (%)*					
18-34 years old	21	28.97 (16.18-41.76)	6716	50.37 (48.74-52.00)	<0.01
35-49 years old	39	71.03 (58.24-83.82)	4985	49.63 (48.00-51.26)	
*Race/Ethnicity (%)*					
Non-Hispanic White	9	29.63 (12.80-46.45)	4974	65.39 (61.86-68.92)	<0.01
Non-Hispanic Black	38	49.13 (32.25-66.01)	2529	11.98 (10.18-13.78)	
Hispanic and others	13	21.24 (7.92-34.57)	4198	22.63 (19.78-25.47)	
*Education (%)*					
<High school	20	28.66 (13.82-43.50)	3170	18.00 (16.64-19.37)	0.17
High school	18	29.35 (16.11-42.59)	2921	24.43 (23.05-25.81)	
>High school	22	41.99 (25.67-58.32)	5602	57.57 (55.54-59.60)	
*PIR (%)*					
<1.0	17	24.93 (12.26-37.59)	2807	15.85 (14.48-17.22)	<0.01
1.0-2.0	17	31.99 (15.20-48.78)	2831	19.02 (17.75-20.29)	
≥2.0	20	33.54 (19.31-47.78)	5301	59.69 (57.65-61.73)	
Missing	6	9.54 (0.66-18.43)	762	5.44 (4.65-6.24)	

Table 3 shows the comparison of geometric means of blood heavy metals by age, gender, race/ethnicity, PIR, and education in subjects with HIV and without HIV. HIV-infected patients whose PIR were <1.00 had significantly higher levels of blood cadmium, lead, and total mercury than non-HIV positive subjects in the same PIR group. In addition, female subjects with HIV infection had higher levels of blood cadmium and lead compared to those females without HIV. Besides, HIV-infected subjects who were male or who were aged from 35 to 49 years old had higher level of blood cadmium than those without HIV. Moreover, HIV-infected patients who were aged from 18 to 34 years old, who reported being neither non-Hispanic white or black, or who had only graduated from high school had higher levels of blood lead compared to those without HIV infections (all p<0.05).

**Table 3:** Comparison of Geometric Means of Blood Heavy Metal Concentration by Demographic and Socioeconomic Status and HIV among US Adults (n=11,761).

	Blood Cadmium (ug/l)					Blood Lead (ug/dl)					Blood Total Mercury (ug/l)				
	HIV		Non-HIV			HIV		Non-HIV			HIV		Non-HIV		
	N	Geometric Mean(95%CI)^a^	N	Geometric Mean(95%CI)^a^	p-value^b^	N	Geometric Mean(95%CI)^a^	N	Geometric Mean(95%CI)^a^	p-value^b^	N	Geometric Mean(95%CI)^a^	N	Geometric Mean(95%CI)^a^	p-value^b^
*Sex*															
Male	47	0.45 (0.35-0.59)	5530	0.32 (0.31-0.33)	<0.01	47	1.46 (1.17-1.82)	5530	1.41 (1.37-1.45)	0.74	47	0.93 (0.59-1.47)	5530	0.95 (0.89-1.01)	0.93
Female	13	0.54 (0.37-0.80)	6171	0.36 (0.35-0.37)	0.03	13	1.34 (0.91-1.96)	6171	0.88 (0.86-0.91)	0.03	13	1.47 (0.66-3.27)	6171	0.87 (0.83-0.92)	0.19
*Age*															
18-34 Years Old	21	0.44 (0.27-0.71)	6716	0.31 (0.30-0.32)	0.17	21	1.41 (0.99-2.01)	6716	0.97 (0.95-1.00)	0.04	21	0.71 (0.52-0.97)	6716	0.80 (0.75-0.85)	0.47
35-49 Years Old	39	0.49 (0.38-0.64)	4985	0.36 (0.35-0.38)	0.03	39	1.44 (1.15-1.81)	4985	1.28 (1.24-1.32)	0.30	39	1.21 (0.71-2.07)	4985	1.04 (0.98-1.11)	0.57
*Race/Ethnicity*															
Non-Hispanic White	9	0.54 (0.33-0.86)	4974	0.33 (0.32-0.35)	0.05	9	1.14 (0.87-1.51)	4974	1.06 (1.03-1.09)	0.58	9	0.81 (0.29-2.27)	4974	0.87 (0.81-0.93)	0.90
Non-Hispanic Black	38	0.50 (0.36-0.71)	2529	0.38 (0.36-0.40)	0.12	38	1.44 (1.11-1.85)	2529	1.17 (1.11-1.24)	0.14	38	0.96 (0.78-1.18)	2529	0.98 (0.91-1.05)	0.85
Hispanic and others	13	0.35 (0.28-0.44)	4198	0.32 (0.31-0.34)	0.50	13	1.95 (1.57-2.41)	4198	1.26 (1.21-1.31)	<0.01	13	1.74 (0.80-3.79)	4198	1.01 (0.94-1.09)	0.16
*Education*															
<High School	20	0.50 (0.34-0.75)	3170	0.42 (0.40-0.45)	0.39	20	1.51 (1.09-2.08)	3170	1.36 (1.31-1.41)	0.53	20	1.54 (0.69-3.44)	3170	0.71 (0.66-0.77)	0.06
High School	18	0.53 (0.37-0.76)	2921	0.39 (0.38-0.41)	0.12	18	1.87 (1.32-2.65)	2921	1.18 (1.14-1.23)	0.01	18	1.21 (0.57-2.55)	2921	0.75 (0.70-0.81)	0.19
>High School	22	0.42 (0.29-0.61)	5602	0.29 (0.29-0.30)	0.06	22	1.15 (0.93-1.41)	5602	1.02 (0.99-1.05)	0.25	22	0.71 (0.47-1.07)	5602	1.06 (1.00-1.13)	0.06
*PIR*															
<1.0	17	0.75 (0.49-1.13)	2807	0.43 (0.40-0.46)	<0.01	17	2.34 (1.60-3.42)	2807	1.21 (1.15-1.28)	<0.01	17	1.00 (0.78-1.28)	2807	0.70 (0.64-0.76)	<0.01
1.0-2.0	17	0.41 (0.28-0.60)	2831	0.37 (0.35-0.38)	0.51	17	1.10 (0.86-1.42)	2831	1.17 (1.12-1.22)	0.64	17	0.85 (0.42-1.72)	2831	0.79 (0.74-0.86)	0.85
>2.0	20	0.41 (0.26-0.64)	5301	0.31 (0.30-0.32)	0.20	20	1.24 (0.96-1.60)	5301	1.06 (1.03-1.09)	0.24	20	1.24 (0.52-2.95)	5301	1.02 (0.96-1.09)	0.65
Missing	6	0.37 (0.20-0.70)	762	0.35 (0.33-0.37)	0.82	6	1.59 (0.99-2.57)	762	1.22 (1.16-1.29)	0.27	6	1.16 (0.42-3.20)	762	0.90 (0.77-1.05)	0.62

^a^ Weighted geometric mean with 95% CI

^b^ p-value obtained from t-test after accounting for the complex survey design.

Table 4 presents the results from multivariate regressions that showed the difference of blood cadmium, lead and total mercury according to HIV infection status after adjusting for age, gender, race/ethnicity, education, PIR, and smoking. The results from model 1 show that HIV status was significantly associated with increased blood cadmium. People with HIV infection have average blood cadmium level 1.16 times (95%CI: 1.01-1.34) higher those without HIV infection (p=0.03). However, similar trends were not observed for blood lead and total mercury between people with and without HIV infection. Finally, we also did the analyses after additionally adjusting for the variable of pack-years of smoking, we observed consistent results. Therefore, we only presented the data from the model without the variable of pack years.

**Table 4:** Relationship between HIV Status and Log-transformed Blood Heavy Metal Concentrations among US Adults (n=11,761).

	Blood Cadmium ^a^		Blood Lead ^a^		Blood Total Mercury ^a^	
	Estimate(95% CI)	p-value	Estimate(95% CI)	p-value	Estimate(95% CI)	p-value
*HIV Status*						
HIV	0.15 (0.01, 0.29)	0.03	-0.05 (-0.23, 0.13)	0.57	0.08 (-0.33, 0.49)	0.69
Non-HIV						
*Age*						
18-34 Years Old						
35-49 Years Old	0.22 (0.19, 0.24)	<0.01	0.32 (0.28, 0.35)	<0.01	0.24 (0.18, 0.29)	<0.01
*Sex*						
Female	0.31 (0.28, 0.33)	<0.01	-0.41 (-0.44, -0.39)	<0.01	-0.12 (-0.16, -0.07)	<0.01
Male						
*Race/Ethnicity*						
Others	0.14 (0.10, 0.18)	<0.01	0.20 (0.16, 0.25)	<0.01	0.29 (0.20, 0.38)	<0.01
Non-Hispanic Black	0.06 (0.02, 0.10)	<0.01	0.11 (0.06, 0.16)	<0.01	0.24 (0.15, 0.34)	<0.01
Non-Hispanic White						
*Education*						
>High School	-0.05 (-0.09, -0.01)	0.02	-0.12 (-0.15, -0.08)	<0.01	0.35 (0.30, 0.41)	<0.01
High School	-0.03 (-0.06, 0.02)	0.20	-0.10 (-0.13, -0.06)	<0.01	0.07 (0.00, 0.13)	0.05
< High School						
*PIR*						
>2.0	-0.04 (-0.08, 0.01)	0.09	-0.06 (-0.10, -0.02)	<0.01	0.26 (0.18, 0.34)	<0.01
1.0-2.0	-0.02 (-0.06, 0.02)	0.32	-0.02 (-0.07, 0.02)	0.34	0.07 (0.00, 0.14)	0.05
Missing	-0.01 (-0.08, 0.05)	0.67	0.02 (-0.04, 0.08)	0.57	0.16 (0.03, 0.29)	0.02
<1.0						
*Serum Cotinine * ^*b*^	0.14 (0.14, 0.15)	<0.01	0.04 (0.04, 0.05)	<0.01	-0.02 (-0.02, -0.01)	<0.01

^a^ Adjusted for age, sex, race/ethnicity, education, PIR, and serum cotinine.

^b^ Concentration of Serum Cotinine was log-transformed.

## Discussion

The evidence on exposure levels to environmental pollutants among HIV-infected populations is very limited. In this study, we found higher body levels of cadmium in persons with HIV. Even after adjusting for pack-years, HIV infections were still marginally associated with blood cadmium which may due to reduced power because of the missing values of pack-years (16% were missing). The observed result could be caused, in part, by generally increased prevalence of exposure to poverty and residing in inner-city areas, which often have higher exposure to environmental pollutants [31–36]. Another possible explanation to higher body levels of heavy metals in person with HIV is that exposures might be similar but HIV infection may interfere with the body’s ability to clear these toxins because HIV infection and the use of ART could impair renal and liver functions, which may lead to impaired detoxification ability and thus further influence the clearance of heavy metals in the body [37–39]. Regardless of the mechanisms, our findings from a nationally representative sample from NHANES demonstrate a significantly higher level of cadmium in persons with HIV and suggest that HIV-infected individuals are potentially at a higher risk of exposure to environmental pollutants. Therefore, persons with HIV represent an at-risk group who may benefit from additional screening for exposure, or intervention strategies to reduce exposure. The findings of this study were consistent with previous findings. A Russian study suggests that persons living with HIV may also extensively be exposed to or accumulate some environmental pollutants (polychlorinated biphenyls, polycyclic aromatic hydrocarbons, lead, and mercury) in the bodies [40]. Another study in different cities of Pakistan also suggested that there was a significant increase in mean values of arsenic, cadmium, nickel, and lead in biological samples of AIDS patients as compared to a controlled healthy male group [41].

The significant difference of heavy metal exposures among HIV-infected subjects may have important clinical implications. Although many studies have shown that the higher rates of noninfectious comorbidities, especially cardiovascular and respiratory diseases, among HIV-infected patients may be primarily due to HIV infection and the use of ART [42–46], the higher exposure to heavy metals suggested in our study may also play an important role in the development of these chronic diseases among HIV-infected patients. The associations between heavy metal exposure and cardiovascular and respiratory diseases have been suggested by many studies [19,47–52]. Furthermore, emerging studies showed heavy metal’s adverse effects on the immune system [53–57]. Therefore, high exposure to these environmental pollutants may aggravate chronic diseases caused by HIV infection and the use of ART, or even initiate these diseases among people with HIV infection. Heavy metal exposure may also help explain the fact that chronic diseases occur at younger age group among this population. It is also possible that adverse effects of these pollutants on immunity may impact the disease progression of HIV but this has not been assessed in the current study [58]. Thus, any synergistic effects between heavy metal exposures and HIV infection remain unknown. Future research should address this question and provide evidence to inform intervention priorities.

Our study has several strengths. The NHANES survey data provide a unique opportunity to compare the exposure levels to environmental pollutants among people with and without HIV infection in a large nationally-representative sample of U.S. adults. In addition, we were able to adjust for many potential confounders including demographic status, socioeconomic status, smoking, and medical condition. Exposure to active and passive smoking was measured by serum cotinine, which is generally regarded as the best choice for quantitative assessment of exposure [27,28,59]. Blood cadmium is reported to have a half-life of 2.5 months and longer [60], which makes it a good indicator of recent cadmium exposure. Furthermore, we also conducted a sensitivity analysis by adjusting for different combinations of selected confounders in the models to test the reliability of our estimation. We found consistent results in these analyses. It suggests that the findings are unlikely observed by chance.

Several limitations in this study should be considered during interpretation of our results. First, the NHANES survey conducted HIV antibody tests mainly to estimate prevalence of HIV infection in the United States population. However, the HIV-infected sample in our study was limited and potentially biased because HIV antibody tests were done only in participants who agreed to the test. Therefore, those who already know or are concerned of their HIV status might refuse and therefore not included in this study. Second, since measurements of many biomarkers for assessing exposure to environmental pollutions (i.e. urinary metabolites of polycyclic aromatic hydrocarbons) were only obtained in a subsample in NHANES, we can only analyze a few of heavy metals with enough sample size in this study. Third, the half-life of lead in human blood is estimated from 28 days to 36 days [61,62], while the half-life of total mercury is around 5.6 days [63]. In addition, although serum cotinine is a well-established biomarker of smoking, its short half-life suggests that it may not be a good indicator of long-term smoking effects. Therefore, they can only reflect recent exposure instead of lifetime exposure if the current exposure sources are greatly different from the past exposure sources. However, this bias is very likely to be non-differential, which may bias the estimation toward null. Fourth, active and/or passive smoking may be an important source of exposure to heavy metals such as cadmium in the general population [49]. Although we included serum cotinine as a biomarker of cigarette smoking in the analysis, this adjustment for cigarette smoking using serum cotinine may not capture long-term effects of cigarette smoking on accumulation of cadmium because of given its short half-life less than a day [64]. Furthermore, although several important potential confounding factors were included in our study, other unselected potential confounders such as occupation and diet may explain some of the difference. Despite limited statistical power given the small sample size, we have generated hypotheses that warrant future examination of heavy metals among HIV infected individuals.

## Conclusion

In summary, our study found that HIV-infected patients have significantly greater exposure to cadmium, compared to those without HIV infection. Considering the limitations of our study, further studies and longitudinal data that collect more pollutants are necessary to determine both the potential increased exposures of a variety of environmental pollutants and their synergistic impacts on chronic disease outcomes in HIV-infected populations. As the life span of HIV-infected patients will continue to increase due to better treatment methods available in future and the number of people living with HIV infection is also expected to be increasing, their exposure to environmental pollution and its effect on their health should receive increased public attention. 
